# Automated detection of knee cystic lesions on magnetic resonance imaging using deep learning

**DOI:** 10.3389/fmed.2022.928642

**Published:** 2022-08-09

**Authors:** Tang Xiongfeng, Li Yingzhi, Shen Xianyue, He Meng, Chen Bo, Guo Deming, Qin Yanguo

**Affiliations:** Department of Orthopaedics, The Second Hospital of Jilin University, Changchun, China

**Keywords:** knee joint, cyst, effusion, magnetic resonance imaging, deep learning

## Abstract

**Background:**

Cystic lesions are frequently observed in knee joint diseases and are usually associated with joint pain, degenerative disorders, or acute injury. Magnetic resonance imaging-based, artificial intelligence-assisted cyst detection is an effective method to improve the whole knee joint analysis. However, few studies have investigated this method. This study is the first attempt at auto-detection of knee cysts based on deep learning methods.

**Methods:**

This retrospective study collected data from 282 subjects with knee cysts confirmed at our institution from January to October 2021. A Squeeze-and-Excitation (SE) inception attention-based You only look once version 5 (SE-YOLOv5) model was developed based on a self-attention mechanism for knee cyst-like lesion detection and differentiation from knee effusions, both characterized by high T2-weighted signals in magnetic resonance imaging (MRI) scans. Model performance was evaluated *via* metrics including accuracy, precision, recall, mean average precision (mAP), F1 score, and frames per second (fps).

**Results:**

The deep learning model could accurately identify knee MRI scans and auto-detect both obvious cyst lesions and small ones with inconspicuous contrasts. The SE-YOLO V5 model constructed in this study yielded superior performance (F1 = 0.879, precision = 0.887, recall = 0.872, all class mAP0.5 = 0.944, effusion mAP = 0.945, cyst mAP = 0.942) and improved detection speed compared to a traditional YOLO model.

**Conclusion:**

This proof-of-concept study examined whether deep learning models could detect knee cysts and distinguish them from knee effusions. The results demonstrated that the classical Yolo V5 and proposed SE-Yolo V5 models could accurately identify cysts.

## Introduction

Benign cysts are frequently encountered during body examinations or advanced knee imaging. Cysts can be categorized into various types, including Baker’s cysts, proximal tibiofibular joint cysts, meniscal cysts, and intraosseous cysts at the insertion of the cruciate ligaments (1). Intra- and periarticular cyst-like lesions are secondary phenomena likely to be observed in painful or osteoarthritis (OA) affected knees (2). They are strongly associated with intra-articular pathologies or complications of various disorders, such as trauma, meniscus injury, infection, inflammatory arthritis, and malignant lesions (3). Cysts and joint effusion are also key features in two semi-quantitative assessments of knee OA, the Whole-Organ Magnetic Resonance Imaging Score (WORMS) and the MRI Osteoarthritis Knee Score (MOAKS) (4, 5). Such fluid accumulation may range from benign to minimally symptomatic and poses a diagnostic dilemma if one is unaware of the potential diagnoses and pitfalls (3). Therefore, it is crucial to develop an appropriate differential diagnosis of knee cystic lesions to guide further evaluation and treatment of OA.

Magnetic resonance imaging is commonly used to confirm whether lesions are cystic due to its superior soft-tissue contrast and multi-planar imaging capabilities compared to other imaging modalities (1). MRI can help delineate the location of lesions concerning anatomic structures and, with the application of contrast, determine if lesions are cystic or solid (6). Typically, cysts located around the knee are encapsulated fluid collections with low T1-weighted signals and high T2-weighted signals on MR scans, similar to benign intra-articular fluid collections, effusions, or certain types of soft-tissue tumors (7–10). Radiologists and clinicians must familiarize themselves with the MRI features of the cyst and cyst-like lesions to accurately diagnose the disease, develop treatment plans, and manage patients more effectively.

Artificial intelligence and deep learning are increasingly utilized in the medical field both in medical imaging and biomedical analysis (11, 12). The role of AI in medical imaging of knee joints has been described in many primary publications (13), with an emphasis on OA-related research, such as auto-segmentation of knee joint tissue (14, 15), and auto-detection of cartilage lesions, meniscus injuries, and anterior cruciate ligament tears (16–19). The deep learning models for such detection demonstrated relatively superb accuracy, ranging between 70 and 100% across various studies, suggesting that such methods exhibit the potential to rival human-level performance in decision-making tasks related to the MRI-based diagnosis of knee injuries. These methods promote the growth of medical enterprises and help create more intelligent medical services.

Most of the current deep learning research on the knee joint focuses on knee OA and acute knee injuries, but few studies have examined knee joint cysts, cyst-like lesions, or joint effusion. In 2018, a deep convolutional neural network (CNN) was applied to the segmentation of knee joint anatomy, achieving dice coefficients between 0.7 and 0.8 for both joint effusion and Baker’s cyst for each joint (20). A more recent study constructed a dense neural network (CNN) for detecting effusions, defined as nonzero MOAKS-ES scores, from limited MRI scans (21). It was demonstrated that NNs could classify knee effusions from low-resolution images with similar accuracy to human radiologists, suggesting that automated evaluation of scans from low-cost, low-field scanners could help assess knee effusions. Other than these two publications, there is no other literature on applying deep learning to cyst detection. It remains unclear whether deep learning techniques can detect cysts and distinguish them from effusions.

Most of the current deep learning research about knee joints focuses on knee osteoarthritis and acute knee injuries, and very few studies examine knee joint cysts, cyst-like lesions, or joint effusion. In 2018, a deep convolutional neural network was applied to the segmentation of knee joint anatomy in a study published by Liu et al. (20). Using the deep learning model, 20 subjects in sagittal frequencies selected fat-suppressed 3D fast spin echo sequences were segmented using 12 different joint structures, and a Dice coefficient between 0.7 and 0.8 was achieved for both joint effusion and Baker’s cyst for each joint. This is the first attempt at deep learning used on joint effusions and cysts. In 2022, Harvard University Bragi Sveinsson carried out a study that created a dense NN (CNN) for detecting effusions, defined as nonzero MOAKS-ES scores, from limited MRI scans (21). Additionally, it was proved that neural networks can classify knee effusions with similar accuracy to that offered by human radiologists utilizing low-resolution images, suggesting that automated assessment of images from low-cost, low-field scanners may be useful for assessing knee effusions. Other than the two publications mentioned above, there are no other literature reports on the application of deep learning to cyst detection. It is not clear whether deep learning technology can be used to detect cysts and the performance of identifying them from effusions.

The present study introduced a deep learning model for the auto-detection of knee cystic lesions to address this knowledge gap. It evaluated the model’s performance in differentiating knee cysts from knee effusions, which could facilitate the early diagnosis and prevention of knee cysts in mass detection by clinicians. To our knowledge, this is the first attempt at automatically detecting knee cysts and distinguishing them from knee effusions using deep learning methods. Because of the limited amount of data, Mosaic augmentation was used in data preprocessing to increase the volume of training data. To enhance the ability to detect cysts of various sizes, Yolo-V5 was used as a backbone network alongside a featured pyramid architecture for detection. An attention mechanism, the SE module, was added to the model to enhance the contribution of information-rich features in the feature extraction process.

## Materials and methods

The Institutional Review Board of the Second Hospital of Jilin University approved this retrospective study (No. SB2021-012).

### Patient data selection

All knee MRIs were acquired at the Second Hospital of Jilin University between January 2021 and October 2021. An in-house RIS/PACS search engine was used to identify candidates who met the following inclusion and exclusion criteria. The inclusion criteria were: (I) MRI scan of the knee for space-occupying lesions or swelling, or pain in a knee joint; (II) patient is over 18 years old; and (III) a formal description of a cystic lesion or uncertain space-occupying lesion in the written radiology report. The exclusion criteria were: (I) patient not consenting to usage of their data; (II) patient is under 18 years old; (III) patient with fracture of a knee joint; (IV) images with excessive movement or beam hardening artifacts as described in the report; and (V) images with knee surgery implants. For patients with more than one MRI examination, only the most recent MR scan was selected.

**FIGURE 1:**
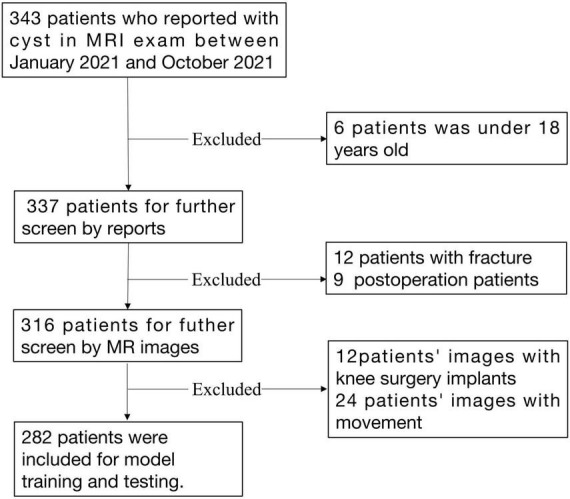
Flowchart of subject inclusion/exclusion and data selection.

Data were retrieved for subjects diagnosed with knee cysts or effusions on the imaging report. If there was uncertainty about including a case, a decision was made after reviewing the original image. A total of 282 cases were included in the final analysis. Patient demographics are listed in Table 1. A detailed data selection flowchart is outlined in Figure 1.

**TABLE 1:** Patient demographics (mean ± s.d.).

Basic information	Total subjects (*n* = 282)	Female (*n* = 192)	Male (*n* = 90)	*P*-value
Age(years)	52.87 ± 13.22	52.95 ± 13.18	52.95 ± 13.24	–
Height(cm)	164.60 ± 7.63	164.19 ± 7.63	164.61 ± 7.64	0.085
Weight(kg)	68.75 ± 11.87	68.75 ± 11.90	68.81 ± 11.90	0.239
BMI(kg/m2)	25.30 ± 3.55	25.30 ± 3.55	25.36 ± 3.73	0.720
Left/Right	143/139	101/91	42/48	–

### Data process

Magnetic resonance imaging was performed on a GE Discovery MR750 3.0T scanner using a sagittal proton density-weighted fat suppression sequence (PD-FS) [Field of view (FOV) = 160 mm × 160 mm; matrix = 512 × 512; number of slices = 20; voxel resolution = 0.35 × 0.35 × 4.5 mm; slice thickness = 3.5 mm; interslice gap = 4.5 mm; repetition time (TR) = 2,600 ms; echo time (TE) = 34.0 ms; flip angle = 90°]. A total of 5,640 sagittal PD-FS images from all subjects were included in this dataset.

Digital Imaging and Communications in Medicine (DICOM) images were converted to one-channel grayscale PNG images to standardize the format of the image files before training. Images were then rescaled to 256 × 256 pixels, and pixel values were normalized between 0 and 1. Two physicians verified that no information related to knee cyst enlargement and effusion was lost in the PNG format images.

**FIGURE 2:**
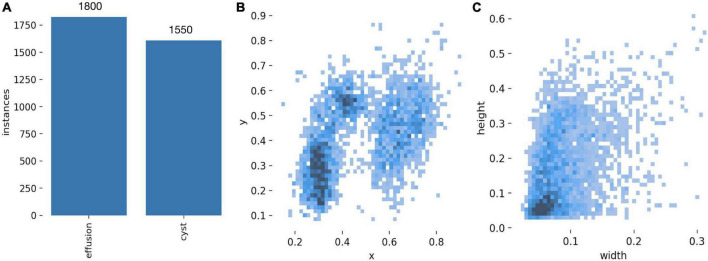
**(A)** Distribution of cyst category. **(B)** Distribution of centroids of cysts and effusions. **(C)** Size distribution of cysts and effusions.

Subsequently, regions of interest (ROIs) of cyst lesions and effusions were annotated using the LabelImg image data annotation software by two resident physicians under the supervision of the chief physician. If the annotation was questionable, the final determination was decided by negotiation with the review panel. Background information surrounding the ROIs was removed whenever possible. Annotation files were stored in Pascal-VOC format during the process. Subsequently, the images and their associated annotation files were divided into a training set, a validation set, and a test set in a ratio of 6:2:2 in the enhanced data set through a Python script. The data distribution of each lesion category and characteristic is shown in Figure 2.

### Deep learning model structure

**FIGURE 3:**
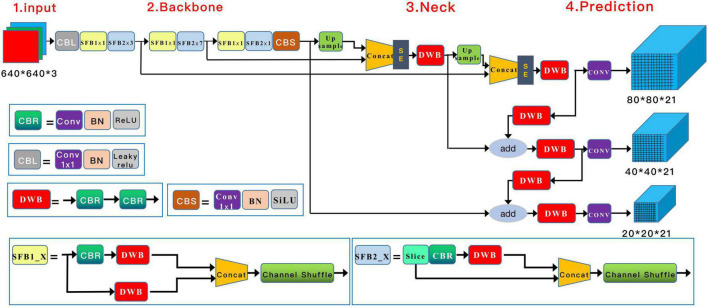
SE-YOLOv5 model architecture for cyst detection.

A Squeeze-and-Excitation (SE) inception attention-based YOLO v5 algorithm (Yolo V5-SE) was adopted to detect knee cyst targets. Similar to the general Yolo v5 algorithm, the architecture of our model was composed of four parts, input, backbone, neck, and prediction, with adjustments in the input and neck parts. In the input preprocessing stage, the images were resized to 640 × 640 × 3, and mosaic data augmentation was applied to increase the number of training samples. Through operations such as flipping, zooming, and color gamut modification, this strategy allowed smaller cyst elements to be detected in a smaller field of sensation, thus enhancing the likelihood of detecting small targets. A couple of SE-inception modules were added after the Concat module in the neck structure (22). The architecture of the model is shown in Figure 3.

### Model training and evaluation

During model training, the learning rate was set to 0.0001 to accelerate model convergence. Stochastic gradient descent (SGD) was used for hyperparameter tuning, and the learning rate momentum was set to 0.90, considering the small number of samples in the cystic lesion dataset. A cosine annealing decay strategy was used for a learning rate change. Cross-entropy was used as the loss function for the model, with batch size set to 8 and training epochs set to 300. The training process was controlled by the early stop method. The training was stopped to prevent over-fitting when the loss value of the validation set did not decrease within 15 epochs. The environment configuration used in the experiment is shown in Table 2.

**TABLE 2:** The environment configuration used in the experiment.

Environment	Detail
Central Processing Unit(CPU)	Intel i7-8700k
Opertating system	Window 10
Graphic Processing Unit(GPU)	NVIDIA Geforce GTX1080i 11G
Pytorch version	Pytorch1.8.1 Opencv 4.5.0

**FIGURE 4:**
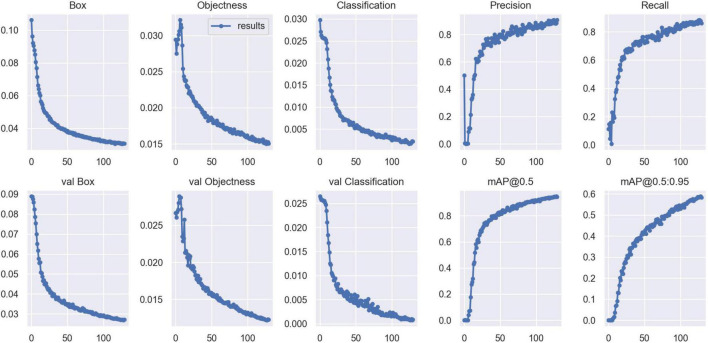
Validation metrics for SE-YOLO V5. The horizontal axis denotes the number of iterations.

Validation metrics, including accuracy, precision, recall, mean average precision (mAP), and F1 score, were calculated and visualized in Python to evaluate model performance in cyst and effusion detection (Figure 4). The formulas for the metrics are described below.


(1) Precision=(TP)/(TP+FP)

(2) Recall=(TP)/(TP+FN)

(3) $$\mathrm{IoU}=\frac{\mathrm{area}\,\mathrm{of}\,\mathrm{overlap}}{\mathrm{area}\,\mathrm{of}\,\mathrm{union}}$$

(4) $$\mathrm{AP}=\int_{0}^{1}P\,\left(R\right)\,\mathrm{dR}$$

(5) $$\mathrm{mAP}=\frac{1}{C}\,\sum_{i}^{C}=1\,A\,P\,\left(i\right)$$

(6) Fscore=2*(}precision*recall)/(}precision+recall)

True positives (TP) denote correctly identified cysts, false positives (FP) denote incorrectly identified cysts, and false negatives (FN) denote missed cysts. AP describes average precision; P(R), which denotes the precision P of different recall rates R, corresponds to the P–R curve’s area under the curve. The constant C in Eq. 5 has a value of 2, representing cysts and effusions as two separate lesions. The number of average precisions (AP) in each category, which is the number of APs in each category when intersection over union (IoU) is 0.5, is denoted as the mean average precision (mAP). Among these metrics, mAP is the most comprehensive index for evaluating model performance, with higher mAP values corresponding to better model performance.

Furthermore, we compared the performance of our Yolo V5-SE model with that of a general Yolo V5 model by comparing the validation metrics. All statistical tests were performed with SPSS Statistics 26.0 (IBM Corp, Armonk, NY, United States).

## Results

Figure 2A shows that the proportion of effusions and cysts was relatively balanced, suggesting that model performance was unlikely to be biased by an imbalanced class distribution. Few lesion centroids were concentrated near the image center, and the distribution of lesion targets was fairly uniform (Figure 2B). Small target lesions accounted for many lesions (Figure 2C).

**FIGURE 5:**
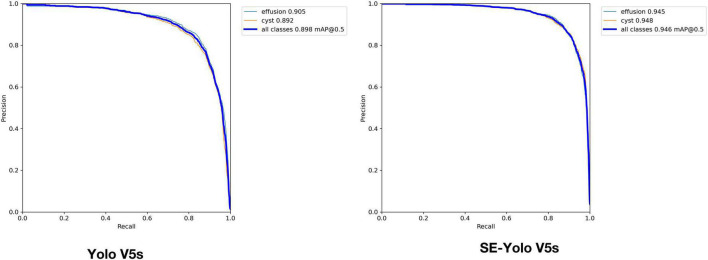
P-R curves of YOLO V5 and YOLO V5-SE.

**FIGURE 6:**
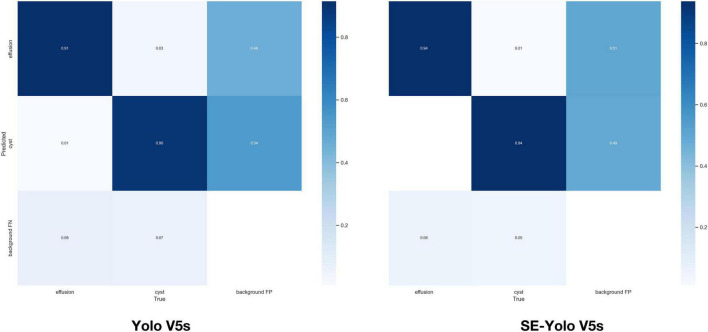
Confusion matrices of YOLO V5 and YOLO V5-SE.

**FIGURE 7:**
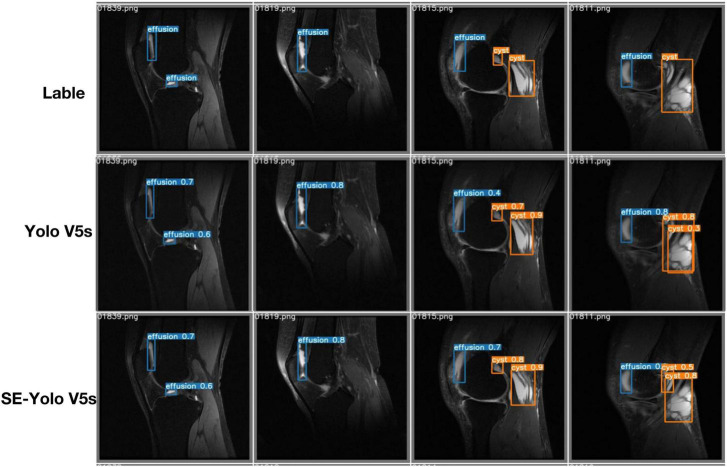
Example prediction outcomes of YOLO V5 and YOLO V5-SE compared with the ground truth.

Validation metrics demonstrated that the model’s performance gradually steadied with the training process, indicating that the model converged quickly and yielded good performance. To assess model performance, our proposed SE model was compared against a classical model, YOLOv5, on a series of performance metrics (Table 3). The SE-YOLO V5 model we presented was superior in all performance metrics (F1 = 0.879, precision = 0.887, recall = 0.872, all class mAP0.5 = 0.944, effusion mAP = 0.945, cyst mAP = 0.942). The fps for SE-Yolo v5 was 90.9, suggesting that it could handle more images per unit time. The P–R curves and confusion matrices for these two models are shown in Figures 5, 6. Figure 7 shows example model prediction results compared to the ground truth, indicating that cyst lesions were correctly detected and distinguished from effusions.

**TABLE 3:** Performance metrics of the SE model and the traditional model.

Metrics	SE-Yolo V5s	Yolo V5s	*P*-value
All class F1 score	0.879 ± 0.002	0.832 ± 0.010	0.002**
All class Precision	0.887 ± 0.011	0.843 ± 0.012	0.011*
All class Recall	0.872 ± 0.014	0.821 ± 0.018	0.018*
All class mAP 0.5	0.944 ± 0.002	0.898 ± 0.011	0.002**
Cyst F1 score	0.875 ± 0.004	0.819 ± 0.016	0.005**
Cyst Precision	0.873 ± 0.012	0.822 ± 0.017	0.014*
Cyst Recall	0.878 ± 0.006	0.818 ± 0.027	0.021*
Cyst mAP 0.5	0.942 ± 0.005	0.893 ± 0.019	0.011*
Effusion F1 score	0.883 ± 0.006	0.843 ± 0.005	0.001**
Effusion Precision	0.902 ± 0.011	0.864 ± 0.008	0.014*
Effusion Recall	0.865 ± 0.022	0.822 ± 0.009	0.037*
Effusion mAP 0.5	0.945 ± 0.001	0.901 ± 0.004	<0.001***

*P < 0.05; **P < 0.01; ***P < 0.001.

## Discussion

This proof-of-concept study aimed to demonstrate the feasibility of a deep learning system for the auto-detection and classification of knee cysts. The SE-YoloV5 attention model was constructed, trained, and evaluated on clinical MR images. Analysis of model performance indicated that this approach promises to improving diagnostic accuracy.

Deep learning offers excellent performance for segmenting multi-tissue knee joints and detecting ACL, cartilage, or meniscus injuries (15–17). However, few papers have addressed cysts and effusions of the knee joint, which are associated with high morbidity and could also serve as biomarkers for degenerative disorders or acute injuries, like knee osteoarthritis and meniscus injuries. Considering the importance and the potential pitfalls of knee cyst diagnosis, it is beneficial to develop an auto-diagnostic system for cyst detection, which may be used as a primary or supplementary tool to speed up diagnosis and enhance accuracy. Two papers have explored the application of deep learning in cyst segmentation and effusion estimation (20, 21); Zhou et al. (20) demonstrated the application of deep learning in Baker’s cyst and joint effusion auto-segmentation and achieved a dice coefficient of 0.736. Raman reported the feasibility of classifying knee effusion based on neural networks, which could achieve an average accuracy of 62%, comparable to a radiologist in a small test dataset (21). Other than the two publications mentioned above, there are no other literature reports on the application of deep learning to cyst detection. To our knowledge, this paper is the first to use deep learning in knee cyst detection.

Cyst detection is an object detection task in nature. Object detection is a primary computer vision task that entails determining where particular objects are in an image and classifying them. YOLO, a new algorithm deployed in 2015 (23), redefined object recognition as a regression problem that can be performed in a single neural network. Yolo has been updated to version five and is regarded as the state-of-the-art algorithm for object detection (24). It has been applied in many daily life aspects, such as the detection of surface knots (25) and real-time vehicles (26), as well as in various medical fields, including face mask recognition (27), breast tumor detection and classification (28), and chest abnormality detection (29). This study showed that the basic deep learning model Yolo V5 could handle the cyst-detection task, attaining F1, precision, and mAP scores of 0.832, 0.843, and 0.821, respectively. After the attention SE module was added to the Yolo V5 model, the resulting attention-based model SE-Yolo V5 achieved better accuracy and higher speed of 0.879, 0.87, and 0.944 for F1 score, precision, and mAP, respectively. Small target lesions accounted for a significant proportion of our dataset, but the proposed model was also capable of detecting them accurately, as illustrated in Figure 7.

This paper aimed to demonstrate the feasibility of utilizing deep learning in general knee cyst detection. Despite its promise, there are several limitations to the presented model. First, there are many cyst types, such as Baker’s cysts, meniscal cysts, and intraosseous cysts at the insertion of the cruciate ligaments, but these different cyst sub-types were not explicitly classified in this study. Neither did we verify whether deep learning performed equally well in these sub-groups. We may enroll more kinds of knee cysts in the future and evaluate the model’s performance on different cyst types. Second, our data was relatively limited, and model performance was not compared with human diagnosis. Nevertheless, the model prediction proved efficient and reliable, suggesting that the model may become a valuable tool for radiologists and clinicians, subject to further study and multi-center validation. Third, the cysts were easily classified based on the reports or images, but there was no general standard for diagnosing inherent effusions, which might be a caveat for the model, radiologist opinions, and the ground truth labels. Last but not least, the uncertainties and interpretability of the model should be mentioned, and we will explore them in further studies. To explore the model in the external datasets or public datasets.

## Conclusion

This proof-of-concept study examined whether deep learning models could detect knee cysts and distinguish them from knee effusions and demonstrated that the classical Yolo V5 and proposed SE-Yolo V5 models could identify cysts with high accuracy. This study suggested that cutting-edge deep learning methods constitute a promising avenue of research to develop AI-assisted auto-detection systems to facilitate radiological and clinical diagnosis of knee pathologies.

## Data availability statement

The raw data supporting the conclusions of this article will be made available by the authors, without undue reservation.

## Ethics statement

The studies involving human participants were reviewed and approved by the local Institutional Review Board (The Second Hospital of Jilin University). Written informed consent for participation was not required for this study in accordance with the national legislation and the institutional requirements.

## Author contributions

TX and QY: conceptualization. TX, HM, and CB: methodology and formal analysis. HM, GD, CB, and SX: validation. HM, GD, and CB: investigation. HM and TX: resources and data curation. TX: writing—original draft preparation. SX and LY: writing—review and editing. QY: supervision and funding acquisition. TX, SX, and QY: project administration. All authors contributed to the article and approved the submitted version.
